# Effect of calcium ions on the aggregation of highly phosphorylated tau

**DOI:** 10.1016/j.bbrep.2024.101887

**Published:** 2024-11-24

**Authors:** Toru Tanaka, Sachiyo Ohashi, Akihiko Takashima, Shunsuke Kobayashi

**Affiliations:** a Laboratory of Biochemistry, School of Pharmacy, Nihon University, Narashinodai, Funabashi, Chiba, 274-8555, Japan; b Laboratory of Alzheimer's Disease, Department of Life Science, Faculty of Science, Gakushuin University, 1-5-1 Mejiro, Toshima-ku, 171-8588, Tokyo, Japan

**Keywords:** Tau, Highly phosphorylation, Calcium, Aggregation, Excitotoxicity

## Abstract

Tau is typically an axonal protein, but in neurons of brains affected by Alzheimer's disease (AD), aggregation of hyperphosphorylated tau in the somatodendritic compartment causes neuronal death. We have previously demonstrated that tau mRNA is transported within dendrites and undergoes immediate translation and hyperphosphorylation of AD epitopes in response to NMDA receptor stimulation. Although this explains the emergence of hyperphosphorylated tau in dendrites, the relationship between tau hyperphosphorylation and aggregation is not well understood. In this study, we found that recombinant highly phosphorylated tau purified from NG108-15 rodent neuroblastoma/glioma cells transfected with both tau and GSK3β expression vectors bound calcium ions and formed sarkosyl-insoluble aggregates. In addition, thioflavin T analysis revealed that this highly phosphorylated tau tended to aggregate on its own, further facilitated by calcium ions. When NG108-15 cells expressing the highly phosphorylated tau were treated with calcium ionophore, sarkosyl-insoluble tau was generated. Interestingly, these cells exhibited resistance to both calcium ionophore-induced cytotoxicity and glutamate-induced excitotoxicity. We further found that sarkosyl-insoluble phosphorylated tau was increased in cultured hippocampal neurons due to glutamate-induced hyperactivity. Our data suggest that hyperphosphorylated tau synthesized in response to NMDA receptor stimulation contributes to regulation of neuronal activity by binding calcium ions, but that this calcium binding may cause tau to adopt an aggregated form.

## Highlights

• Highly phosphorylated tau binds calcium ions to phosphate groups on the molecule.
• Calcium ions induce the formation of sarkosyl-insoluble tau aggregates.
• Calcium-bound phosphorylated tau forms aggregates containing β-sheet structures.
• Sarkosyl-insoluble phosphorylated tau is increased by NMDA receptor stimulation.
• Calcium binding of highly phosphorylated tau attenuates excitotoxicity.

## 1 Introduction

In healthy neurons, tau is distributed mainly in the axon and plays a role in polymerization and stabilization of microtubules. However, in neurons of brains affected by Alzheimer's disease (AD), hyperphosphorylated tau forms insoluble neurofibrillary tangles (NFTs) and accumulates prominently in somatodendrites [1]. NFTs are a pathological hallmark of AD along with senile plaques composed of extracellular deposits of amyloid β (Aβ), which induces tau aggregation [2], and propagation of NFTs from the entorhinal cortex to the neocortex is well correlated with progression of the disease [3]. Indeed, the toxicity associated with aggregation of tau from an oligomer to NFTs results in loss of synapses and, subsequently, neurons [4]. Therefore, to further clarify the pathogenesis of Alzheimer's disease, it is important to characterize the process by which hyperphosphorylated tau emerges locally in dendrites and aggregates there.

Regarding the appearance of tau in dendrites of AD neurons, we have recently reported that tau mRNA and mRNAs for GSK3β and CDK5, known to be major tau kinases, are distributed in dendrites and that hyperphosphorylated tau with AD epitopes is locally synthesized in response to NMDA receptor-mediated stimulation [[5], [6], [7]]. In the present study, we investigated the mechanism responsible for aggregation of hyperphosphorylated tau.

Glutamatergic stimulation of the NMDA receptor causes a high concentration of calcium to enter synapses, thus activating neurotransmission, and during this process hyperphosphorylated tau is synthesized. We found that phosphate groups on the tau molecule became bound to calcium ions. This calcium-bound highly phosphorylated tau formed sarkosyl-insoluble aggregates containing β-sheet structures *in vitro*. Sarkosyl-insoluble tau was also obtained as a result of calcium influx into NG108-15 cells expressing highly phosphorylated tau. Interestingly, such cells showed resistance to calcium-induced cytotoxicity and NMDA receptor-mediated excitotoxicity. We also demonstrated that in cultured hippocampal neurons, sarkosyl-insoluble phosphorylated tau was obviously increased by NMDA receptor-mediated stimulation. These results suggest that although hyperphosphorylated tau may play a neuroprotective role against hyperactivity by binding calcium ions to phosphate groups on the molecule, this calcium binding leads to undesirable tau aggregation.

## 2 Materials and Methods

### 2.1 Animal experiments

Hippocampal neurons were prepared from embryos (E18) obtained from two pregnant C57BL/6J mice. Animal experiments were performed under a protocol approved by the Ethics Committee for Animal Experimentation of Nihon University (approval number; AP21PHA016-1).

### 2.2 Antibodies and agents

Anti-tau (mouse) monoclonal antibody Tau-5 (catalog no. ab80579, lot no. GR3266691-6) and anti-tau pSer199 (rabbit) monoclonal antibody (catalog no. ab81268, lot no. GR3228846-1) were from Abcam. Anti-tau pThr231 (mouse) monoclonal antibody (AT180) (catalog no. MN1040, lot no. ZC4211943) and anti-AT8 (mouse) monoclonal antibody (catalog no. MN1020, lot no. PD199773) were purchased from Thermo Fisher Scientific. Anti-tau pSer396 (rabbit) monoclonal antibody (catalog no. BS4196, lot no. CJ36131) was obtained from Bioworld Technology. Anti-Human Tau/Repeat Domain (2B11) Mouse IgG MoAb (catalog no. 10237, lot no. 1A-529) was from Immuno-Biological Laboratories. Alexa Fluor 488 donkey anti-rabbit IgG (catalog no. R37118, lot no. 2000234) and Alexa Fluor 488 donkey anti-mouse IgG (catalog no. R37114, lot no. 2000234) were from Invitrogen. Alexa Fluor 555 goat anti-rabbit IgG (catalog no. A21428, lot no. 1937183), glutamate, 4′,6-diamidino-2-phenylindole (DAPI), EGTA and CaCl_2_ were obtained from Wako Pure Chemical Industries. Calf intestine alkaline phosphatase (CIAP) was from Toyobo. Calcium ionophore A23187 and thioflavin T were purchased from Sigma-Aldrich. Metalloassay calcium LS (CPZIII) was from Metallogenics.

### 2.3 Expression and preparation of recombinant highly phosphorylated tau

In order to construct a tau expression vector, the EGFP gene of the pEGFP-C1 vector was excised with AgeI and EcoRI, and human 2N4R-tau cDNA [8,9] was inserted downstream of the CMV promoter instead. A deletion mutant tau lacking the repeat domain (ΔRepeat-tau) was produced by using 2N4R-tau cDNA [9]. As the GSK3β expression vector, human GSK3β cDNA was cloned from SH-SY5Y cells and recombined instead of the EGFP gene. NG108-15 cells were co-transfected with these plasmids using X-tremeGENE HP (Roche), and after overnight culture the cells were lysed in TN buffer containing 20 mM Tris-HCl (pH 7.5), 150 mM NaCl, 1 % Triton X-100, cOmplete ULTRA Tablets (Roche, Protease Inhibitor Cocktail), and Phosphatase Inhibitor Cocktail Solution Ⅰ (FUJIFILM). The lysate was centrifuged at 8700×*g* for 10 min to obtain the post-nuclear supernatant. For purification of highly phosphorylated tau, we used 6 × His-tagged 2N4R-tau expression vector and a HisTrap Column (Ni-NTA chromatography). The sequences of the PCR primer pairs used were as follows.

2N4R-tau:

Forward, 5′-ACCGGTCGCCACCATGGCTGAGCCCCGCCAGGAGTTCGAAG-3′.

Reverse, 5′-GAATTCTCACAAACCCTGCTTGGCCAGGGAG-3′.

6 × His-Tagged 2N4R-tau:

Forward, 5′-ACCGGTCGCCACCATGCATCATCATCATCATCATATCGAAGGTAGG GATGGCTGAGCCCCGCCAGGAGTTCGAAG -3′

Reverse, 5′-GAATTCTCACAAACCCTGCTTGGCCAGGGAG-3′

GSK3β:

Forward, 5′-ACCGGTCGCCACCATGGCTGAGCCCCGCCAGGAGTTCGAAG-3′

Reverse, 5′-GAATTCTCACAAACCCTGCTTGGCCAGGGAG-3′

### 2.4 Cell culture and agent treatment

NG108-15 cells were grown in Dulbecco's modified Eagle medium with 10 % fetal bovine serum and transfected with the tau and GSK3β expression vectors. For calcium ionophore treatment, cells were incubated with 5 μM calcium ionophore A23187 for 5 h at 37 °C in growth medium containing 2 mM CaCl_2_. To examine the effect of highly phosphorylated tau on cytotoxicity, cells were incubated with 2.5 μM calcium ionophore A23187 overnight at 37 °C. To examine the effect on glutamate-induced excitotoxicity, cells differentiated with 1 mM dibutyryl cAMP for 2 days were transfected with expression vectors and then treated with 200 μM glutamate overnight. Differentiated hippocampal neurons were prepared from embryonic (E18) C57BL/6J mice as described previously [7] and exposed to 0.5 mM glutamate for 2 h. For analysis of solubility in sarkosyl solution, proteins or cell extracts were incubated in a 1 % sarkosyl solution at 37 °C for 1 h, and then centrifuged at 21,800×*g* for 15 min. The resulting supernatant or precipitate was analyzed by Western blotting against tau antibodies.

### 2.5 Analysis of calcium binding to highly phosphorylated tau

Preparation of highly phosphorylated tau-binding beads was carried out using Dynabeads Protein G (Life Technologies). The beads were washed with PBS containing 0.1 % BSA and bound to anti-Human Tau/Repeat Domain (2B11) Mouse IgG MoAb (6 μg). They were then incubated with the post-nuclear supernatant (1.5 mg protein) from NG108-15 cells expressing highly phosphorylated tau at 4 °C for 2 h in TN buffer. After washing with TN buffer, the sample was divided into two halves, one of which was treated with CIAP, and each specimen was incubated in 1 mM CaCl_2_ solution at 37 °C for 30 min, collected with a magnet, and then the amount of calcium ions in the supernatant was assayed using Metalloassay calcium LS (CPZIII). For analysis of calcium binding to highly phosphorylated tau in NG108-15 cells, we treated the cells with calcium ionophore, immunoprecipitated cell extracts with anti-tau antibody, and directly analyzed calcium ions in the resulting precipitates. Briefly, we measured the amounts of control 2N4R-tau and highly phosphorylated 2N4R-tau synthesized in NG108-15 attached to tau antibody-coupled beads (Supplementary data 1) and quantified the amount of calcium bound per tau molecule in each case.

### 2.6 Western blot analysis

Proteins were separated by SDS-PAGE and transferred to a PVDF membrane. After treatment with a specific antibody for tau or phosphorylated tau, the membrane was incubated with the second antibody conjugated with alkaline phosphatase (Promega), and the signals were detected with 1-Step NBT/BCIP substrate (Thermo Scientific). For phosphatase treatment, proteins were incubated with CIAP.

### 2.7 Immunocytochemistry

Cells were fixed with 4 % paraformaldehyde in PBS for 15 min and then permeabilized with 1 % Triton X-100 in PBS for 15 min. After blocking with DMEM-containing FBS, the cells were incubated with primary antibody at room temperature for 2 h. After washing with PBS, specimens were incubated with the second antibody at room temperature for 1 h and examined using an Olympus IX73 fluorescence microscope linked to a DP-74 imaging system. In order to analyze the quantitative change and phosphorylation degree of tau in dendrites, measurement of fluorescence intensity was performed using NIH ImageJ software. Data used for comparison were acquired using the same exposure time and sensitivity, and raw fluorescence images were used for quantification. The entire range of each dendrite (MAP2-positive neurite) was specified, the fluorescence intensity per unit area was measured, and the value was obtained by subtracting the fluorescence intensity per unit area of the portion just outside the dendrite as the fluorescent signal. Each fluorescent signal was normalized by the fluorescence intensity of nuclear DAPI staining and the average of the normalized values was used.

### 2.8 Thioflavin T assay

Tau aggregation was determined using thioflavin T. Purified recombinant highly phosphorylated tau (2 μM) and thioflavin T (20 μM) were mixed in buffer containing 20 mM Tris-HCl (pH 7.5) and 150 mM NaCl. After adding CaCl_2_ to a final concentration of 0, 0.1 or 1 mM, the solutions were incubated in a damp box at 37 °C. At each time point indicated in Fig. 3, fluorescence levels were measured at an excitation wavelength of 444 nm and an emission wavelength of 485 nm.

### 2.9 MTT assay

MTT assay was performed to examine the effect of highly phosphorylated tau on calcium ionophore-induced cytotoxicity on undifferentiated NG108-15 cells or glutamate-induced excitotoxicity on differentiated NG108-15 cells in accordance with the standard protocol [10].

### 2.10 Statistical analysis

All data are presented as the mean and standard error. Data were analyzed by Student's *t*-test or one-way ANOVA, followed by Tukey-Kramer post hoc test and differences at *P* < 0.05 were considered to be statistically significant.

## 3 Results

### 3.1 Highly phosphorylated tau binds calcium ions

**Fig. 1:**
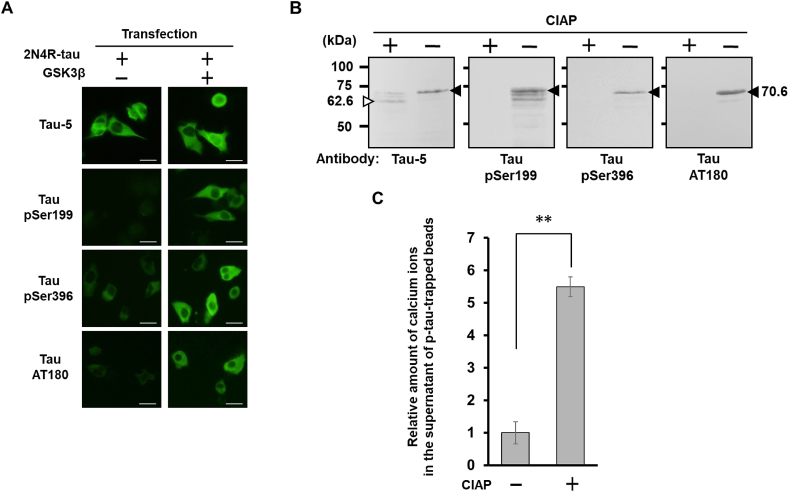
Highly phosphorylated tau binds calcium ions. (A) Synthesis of recombinant highly phosphorylated tau in NG108-15 cells. Cells co-transfected with 6 × His-tagged 2N4R-tau expression vector and GSK3β expression vector were immunostained with anti-tau (Tau-5), anti-tau pSer199, anti-tau pSer396 or anti-tau pThr231 (AT180) antibody. Scale bar: 10 μm. (B) Western blot analysis of highly phosphorylated tau synthesized in NG108-15 cells. Recombinant highly phosphorylated tau was purified using Ni-NTA agarose from CIAP-treated or -untreated cytosolic fractions and analyzed by Western blotting using anti-tau (Tau-5), anti-tau pSer199, pSer396 antibody or anti-tau pThr231 (AT180) antibody. (C) Analysis of the calcium binding property of highly phosphorylated tau. CIAP-treated and -untreated highly phosphorylated tau-trapped beads were incubated in CaCl_2_ solution, and the relative amounts of calcium ions in each supernatant were compared. The data represent the mean and standard error obtained from three independent experiments. *∗∗P* < 0.01 (Student's *t*-test).

We first investigated whether highly phosphorylated tau became bound to calcium ions. To obtain highly phosphorylated tau synthesized in living cells, NG108-15 cells were co-transfected with two expression vectors of the 6 × His-tagged longest human isoform 2N4R-tau and human GSK3β. Intracellular tau phosphorylation was confirmed by immunostaining with anti-phosphorylated tau antibodies for GSK3β epitopes pSer199, pSer396 and pThr231 (Fig. 1). This anti-pThr231 antibody (AT180) is widely used to detect pathological phosphorylated tau. The tau was purified with a nickel affinity column (Ni-NTA agarose) from CIAP-treated or -untreated cell extracts and analyzed by Western blotting using anti-tau antibody Tau-5 that recognizes total tau, and antibodies for phosphor-tau pSer199, pSer396 or pThr231 (Fig. 1B). Phosphorylated tau was detected at a significantly higher molecular mass position (70.6 kDa) than dephosphorylated tau (62.6 kDa), indicating that the highly phosphorylated tau was available. These results indicate that recombinant 2N4R-tau in NG108-15 cells is highly phosphorylated like pathological tau.

The recombinant highly phosphorylated tau was trapped on Protein-G beads to which anti-tau antibody (Anti-Human Tau/Repeat Domain (2B11) Mouse IgG MoAb) was adsorbed. After incubation in 5 mM CaCl_2_ solution, the beads were collected with a magnet, and then using Metalloassay calcium LS (CPZIII), the calcium ions in the supernatant were quantified as the absorbance at 690 nm. In parallel, highly phosphorylated tau-adsorbed beads were dephosphorylated by CIAP treatment, and the same procedure as that described above was performed to compare the relative amount of calcium ion binding (Fig. 1C). The amount of calcium ions in the supernatant was significantly increased by the CIAP treatment. The data show that highly phosphorylated tau became bound to calcium ions at the phosphate groups on the molecule.

### 3.2 Formation of sarkosyl-insoluble aggregates by calcium-bound phosphorylated tau

**Fig. 2:**
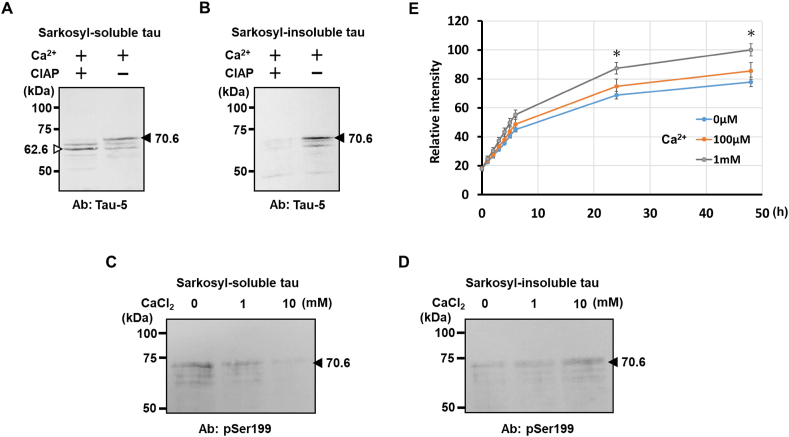
Formation of sarkosyl-insoluble aggregates of highly phosphorylated tau upon calcium binding. (A and B) CIAP-treated and -untreated highly phosphorylated tau (5 μM each) were incubated in 1 mM CaCl_2_ solution. After centrifugation, the resulting precipitate was incubated in 1 % Sarkosyl solution, separated into Sarkosyl-soluble fraction (A) and -insoluble fraction (B) by centrifugation again, and Western blot analysis was performed using anti-tau antibody Tau-5. (C and D) Calcium-dependent sarkosyl-insoluble highly phosphorylated tau aggregation. Highly phosphorylated tau (5 μM) incubated with 0, 1 or 10 mM CaCl_2_ was separated into a sarkosyl-soluble supernatant (C) and a sarkosyl-insoluble precipitate (D) as described above, and analyzed by Western blotting using anti-tau pSer199 antibody. (E) Thioflavin T-binding of the highly phosphorylated tau aggregates in the presence of calcium. A mixture of recombinant highly phosphorylated tau (2 μM) and CaCl_2_ (0, 0.1 or 1 mM) was incubated in the presence of thioflavin T (20 μM), and the change in fluorescence intensity was measured at the indicated time point. Data represent the mean and standard error of three independent experiments with 10 replicates for each calcium concentration. ∗*P* *<* 0.05 versus control (one-way ANOVA, followed by Tukey-Kramer post hoc test).

It has been reported that sarkosyl-insoluble tau aggregation is associated with neuronal death [11]. To examine the ability of calcium-bound phosphorylated tau to form sarkosyl-insoluble aggregates, the recombinant highly phosphorylated tau was split into two halves (5 μM each), one of which was treated with CIAP and the other untreated. Each sample was incubated in 1 mM CaCl_2_ solution at 37 °C for 3 h and centrifuged at 21,800×*g* for 10 min. The resulting precipitate was suspended and incubated in 1 % sarkosyl solution at 37 °C for 1 h, and centrifuged again to obtain a sarkosyl-soluble supernatant and a sarkosyl-insoluble pellet. The distribution of highly phosphorylated tau and dephosphorylated tau to each fraction was then analyzed by Western blotting. Both types of tau were detected in the sarkosyl-soluble fraction (Fig. 2A). On the other hand, in the sarkosyl-insoluble fraction, highly phosphorylated tau, but not dephosphorylated tau, was distributed (Fig. 2B). These results suggested that although tau aggregated in the presence of calcium ions with or without phosphorylation, sufficient phosphorylation was required for the formation of sarkosyl-insoluble precipitates.

**Fig. 3:**
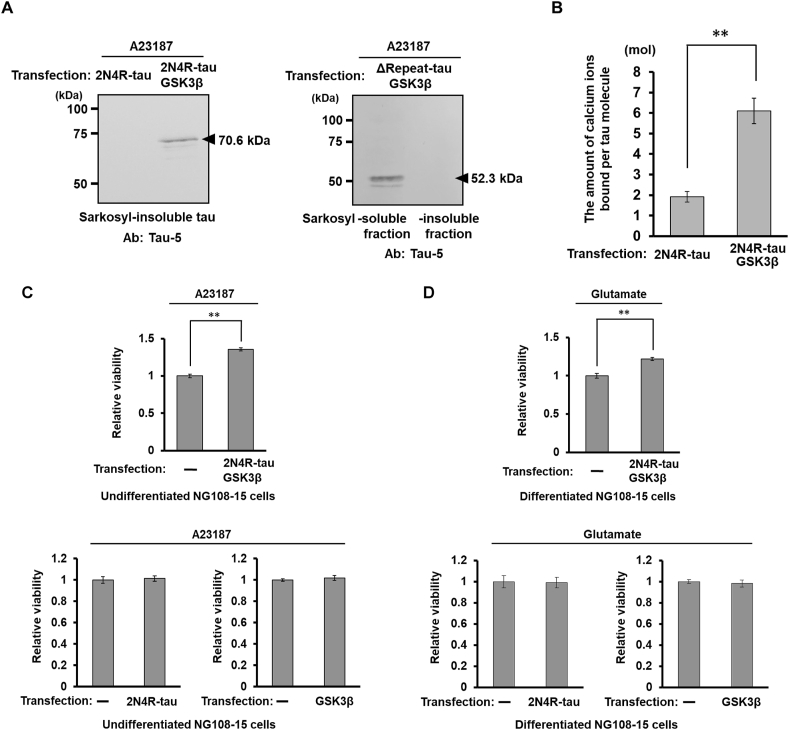
Calcium binding by highly phosphorylated tau in living cells attenuates excitotoxicity. (A) Left panel: NG108-15 cells co-transfected with the 2N4R-tau expression vector and GSK3β expression vector were treated with 5 μM calcium ionophore A23187 for 5 h in culture medium. As a control, cells transfected with 2N4R-tau vector alone were used. Cytosolic fractions were prepared and incubated in a final concentration of 1 % sarkosyl for 1 h at 37 °C, followed by centrifugation at 21,800×*g* for 15 min and the precipitated aggregates were analyzed by Western blotting using an anti-tau antibody Tau-5. Right panel: Cells transfected with both the ΔRepeat-tau and GSK3β vectors were treated with A23187, the cytosolic fraction was centrifuged at 21,800×*g* for 15 min, and the solubility of the ΔRepeat-tau contained in the precipitate in 1 % sarkosyl solution was examined. (B) NG108-15 cells transfected with both 2N4R-tau and GSK3β expression vectors or 2N4R-tau expression vector alone were treated with A23187 as described in (A). Highly phosphorylated tau and control tau were then immunoprecipitated from the post-nuclear supernatant, and the amount of bound calcium was quantified. The amount of tau bound to the beads was then examined (Supplementary data 1) and the molecular ratio of tau to calcium was determined. The data represent the mean and standard error for three independent experiments. ∗∗*P* < 0.01 (Student's *t*-test). (C and D) The effect of highly phosphorylated tau expression on calcium ionophore-induced cytotoxicity on undifferentiated NG108-15 cells (C) or glutamate-induced excitotoxicity on differentiated NG108-15 cells (D) was examined as described in Materials and Methods, and cell viability was measured by MTT assay. Values were normalized by the survival rate of untransfected cells after treatment with A23187 or glutamate. The effects of expression of 2N4R-tau alone or GSK3β alone were also examined (each lower panel). The data represent the mean and standard error for five independent experiments. *∗∗P* < 0.01 (Student's *t*-test).

We then investigated the necessity of calcium ions for formation of sarkosyl-insoluble aggregates composed of highly phosphorylated tau. Purified highly phosphorylated tau treated with different concentrations of CaCl_2_ was centrifuged, and the resulting precipitate was analyzed for sarkosyl solubility as described above. Western blot analysis showed that sarkosyl-insoluble highly phosphorylated tau was apparently increased in a calcium concentration-dependent manner (Fig. 2C and D). Together with the results shown in Fig. 1, it was evident that calcium-bound highly phosphorylated tau formed sarkosyl-insoluble aggregates.

The aggregated tau found in the brains of Alzheimer's disease patients is known to have a β-sheet structure, and tau aggregation can be monitored *in vitro* by measuring changes in the fluorescence intensity of thioflavin T binding to the β-sheet structure [12]. We therefore investigated the thioflavin T-binding ability of the highly phosphorylated tau aggregates in the presence of calcium ions. Binding of thioflavin T to the aggregates was increased relative to that in the absence of calcium, suggesting that calcium ions can contribute to the formation of aggregates containing β-sheet structures (Fig. 2E). However, an increase of fluorescence intensity was observed even in the absence of calcium ions, suggesting that highly phosphorylated tau tends to aggregate on its own, and is further facilitated by calcium binding. This is consistent with a previous report that hyperphosphorylated tau becomes prone to aggregation and causes cell death [13]. Taken together, these findings indicate that toxic aggregates are likely to form within dendrites, where hyperphosphorylated tau increases and an influx of calcium ions occurs upon NMDA receptor stimulation.

### 3.3 Calcium binding of highly phosphorylated tau in living cells attenuates excitotoxicity

To investigate whether sarkosyl-insoluble aggregates of highly phosphorylated tau are formed in living cells, we treated NG108-15 cells overexpressing both 2N4R-tau and GSK3β with calcium ionophore A23187 for 5 h in DMEM containing 2 mM CaCl_2_ and then prepared a sarkosyl-insoluble fraction from the cell extract. As a control, cells transfected with 2N4R-tau alone were also treated in the same manner. Western blot analysis using an anti-tau antibody Tau-5 revealed that in NG108-15 cells expressing highly phosphorylated tau, sarkosyl-insoluble aggregates composed of 70.6-kDa phosphorylated tau were formed, whereas no such aggregates were formed in the control cells (Fig. 3A left panel). Furthermore, when tau lacking the repeat domain (ΔRepeat-tau), which is involved in formation of the β-sheet structure required for tau aggregation, was co-expressed with GSK3β in NG108-15 cells and treated with calcium ionophore, this 52.3-kDa mutant tau was abundantly recovered in the sarkosyl-soluble fraction but was barely detectable in the sarkosyl-insoluble fraction (Fig. 3A right panel). These data suggested that highly phosphorylated tau is able to bind calcium ions in living cells and aggregate via the β-sheet structure. In fact, highly phosphorylated 2N4R-tau obtained from calcium ionophore-treated cells bound approximately 3.2 times more calcium ions than control 2N4R-tau (Fig. 3B and Supplementary data 1).

During these experiments, we noticed that NG108-15 cells overexpressing both 2N4R-tau and GSK3β showed attenuation of the cytotoxicity caused by calcium ionophore treatment. To confirm this phenomenon, cells treated with A23187 overnight were subjected to the MTT assay (Fig. 3C, upper panel). In comparison to untransfected cells, the cells expressing highly phosphorylated tau showed resistance to cytotoxicity caused by calcium ions. Expression of tau alone or GSK3β alone did not confer any cytoprotective effect (Fig. 3 C, lower panels). Intriguingly, this suggested that in neurons, highly phosphorylated tau may trap some of the calcium ions flowing into the cells, reducing the extent of excitotoxicity. Since differentiated NG108-15 cells express the functional NMDA receptor [14,15] and undergo glutamate-induced excitotoxicity [16], we investigated the effects of highly phosphorylated tau on cell viability. Expression of highly phosphorylated tau in differentiated NG108-15 cells attenuated glutamate-induced excitotoxicity (Fig. 3D, upper panel), but not in cells overexpressing tau alone or GSK3β alone (Fig. 3 D, lower panels).

### 3.4 Glutamatergic stimulation induces the formation of sarkosyl-insoluble phosphorylated tau in differentiated hippocampal neurons

**Fig. 4:**
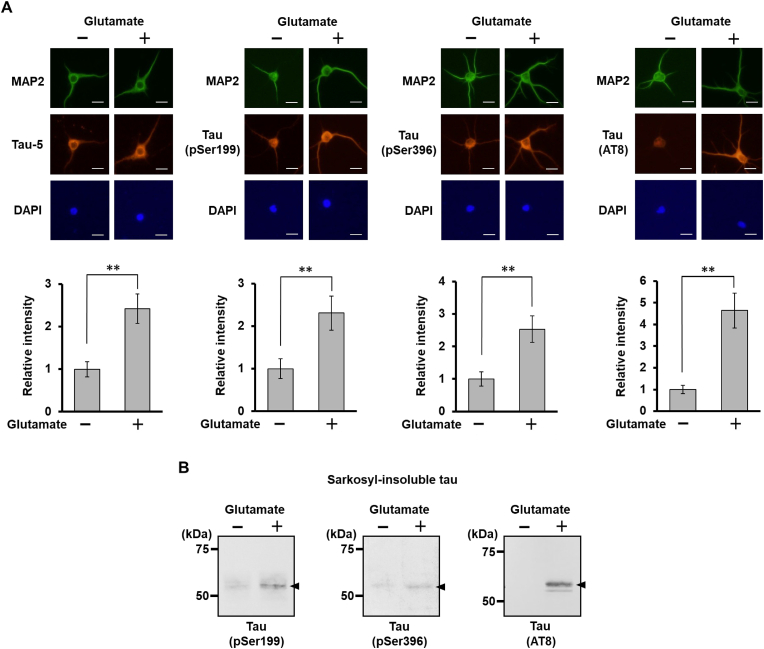
Glutamatergic stimulation-responsive increase of sarkosyl-insoluble phosphorylated tau in hippocampal neurons. (A) Differentiated hippocampal neurons were treated with 0.5 mM glutamate for 2 h and analyzed by immunocytochemistry with anti-tau (Tau-5), anti-tau pSer199, anti-tau pSer396 or anti-AT8 antibody, and anti-MAP2 antibody as a dendritic marker. Scale bar: 10 μm. Quantitative analysis of total tau or each phosphorylated tau in dendrites is shown below each of the immunocytochemical data. Relative tau protein levels (fluorescence intensity) in dendrites of glutamate-treated and untreated hippocampal neurons are shown. Approximately 30 neurons were evaluated. The signal intensities were measured using the NIH ImageJ software package and normalized to the fluorescence intensity of each corresponding DAPI-stained neuron. Data are presented as the mean and standard error. ∗∗*P <*0.01 (Student's *t*-test). (B) Sarkosyl-insoluble fractions were prepared from glutamate-treated and -untreated neurons as described in Fig. 3A, and phosphorylated tau was detected by Western blotting using anti-tau pSer199, anti-tau pSer396 or anti AT8 antibody.

Within dendrites of hippocampal neurons, glutamatergic stimulation induces the synthesis of hyperphosphorylated tau – similar to that observed in AD brain neurons – within 30 min [[5], [6], [7]]. Therefore, using cultured hippocampal neurons, we further investigated whether sarkosyl-insoluble aggregates composed of endogenous hyperphosphorylated tau are generated in response to glutamatergic stimulation. Differentiated neurons were treated with 0.5 mM glutamate for 2 h and immunostained with anti-tau (Tau-5), anti-tau pSer199, anti-tau pSer396 or anti-AT8 antibody – a known AD-relevant epitope – and anti-MAP2 antibody as a dendritic marker. Comparison of the fluorescence intensity in dendrites confirmed that the increase of hyperphosphorylated tau persisted in dendrites even after 2 h of glutamate stimulation (Fig. 4A). Sarkosyl-insoluble precipitates were prepared from these neurons and analyzed by Western blotting using antibodies against phosphorylated epitopes (Fig. 4B). Sarkosyl-insoluble tau was obviously increased in the glutamate-treated neurons. This result confirmed that hyperphosphorylated tau generated in dendrites in response to glutamate stimulation forms sarkosyl-insoluble aggregates.

Taken together, the data obtained in the present study suggest that although hyperphosphorylated tau synthesized in response to NMDA receptor-mediated stimulation contributes to modulation of neuronal activity by binding calcium ions, this calcium-bound phosphorylated tau is prone to formation of aggregates that cause neurodegeneration.

## 4 Discussion

Tau aggregation *in vitro* is induced by the presence of polyanions such as heparin and nucleic acids [12]. Furthermore, it has been reported that tau is contained in stress granules and that the RNA-binding protein TIA1 regulates the generation of toxic tau oligomers [17]. However, the mechanism responsible for aggregation of hyperphosphorylated tau is not well understood.

We have previously reported a mechanism by which hyperphosphorylated tau is generated in dendrites in response to stimulation of NMDA receptors, which are calcium-permeable ion channels [5]. It has been shown that calcium ions precipitate many phosphorylated proteins, including tau, extracted from AD brains [18], and that calcification and hyperphosphorylated tau are colocalized in AD [19]. In the present study, therefore, we investigated the calcium-binding properties of recombinant highly phosphorylated tau and the aggregation properties of the calcium-bound tau. Interestingly, our results showed that highly phosphorylated tau formed aggregates with β-sheet structures by binding to calcium ions in the absence of polyanions.

Glutamatergic stimulation of NMDA receptors causes calcium influx into synapses, activating various reactions involved in signal transduction, but excessive excitation leads to cell death. In this regard, it is intriguing that in our experiments, the presence of highly phosphorylated tau attenuated the degree of calcium-induced excitotoxicity. This may be one of the reasons why hyperphosphorylated tau is increased in dendrites in response to glutamatergic stimulation. Physiological functions of tau other than its role as a microtubule-associated protein include LTD involvement in synapses [20]. In addition, our results suggest that tau undergoes local translation and hyperphosphorylation through strong glutamatergic stimulation and binds to a proportion of calcium ions in synapses, thereby contributing to signal transduction control and synaptic protection from excitotoxicity. On the other hand, however, calcium-bound hyperphosphorylated tau is susceptible to formation of toxic aggregates. This means that functional hyperphosphorylated tau undergoes transformation into an undesirable molecule. Such aggregates need to be eliminated promptly, but in AD brains they may persist due to malfunction of the protein degradation system. An association between autophagy dysfunction and tau pathology has been reported [21,22]. Failure to prevent the formation of toxic tau oligomers and aggregates would lead to neuron death. A scenario in which hyperphosphorylated tau, produced to regulate neuronal activity, binds to calcium but forms toxic aggregates, would support a report that neuronal hyperactivity in MCI patients accelerates progression to AD [23]. It has also been reported that neuronal activity enhances tau pathology [24].

Although details of the aggregation mechanism and function of hyperphosphorylated tau remain to be clarified, it is possible that interaction between calcium phosphate on tau molecules may lead to aggregation.

## 5 Conclusion

It is possible that hyperphosphorylated tau, which is synthesized at synapses in response to NMDA receptor stimulation, binds calcium ions and contributes to the regulation of neuronal activity, but that calcium-bound phosphorylated tau tends to form undesirable aggregates.

## CRediT authorship contribution statement

**Toru Tanaka:** Writing – review & editing, Methodology, Investigation, Data curation, Conceptualization. **Sachiyo Ohashi:** Investigation. **Akihiko Takashima:** Writing – review & editing. **Shunsuke Kobayashi:** Writing – review & editing, Writing – original draft, Visualization, Project administration, Methodology, Investigation, Conceptualization.

## Declaration of competing interest

The authors declare that they have no known competing financial interests or personal relationships that could have appeared to influence the work reported in this paper.
