# Supplementary figures and images for: Effect of calcium ions on the aggregation of highly phosphorylated tau
Source: Biochem Biophys Rep. 2024 Nov 24;40:101887. doi: 10.1016/j.bbrep.2024.101887 (PMC11626071; doi:10.1016/j.bbrep.2024.101887)

## Slide 1
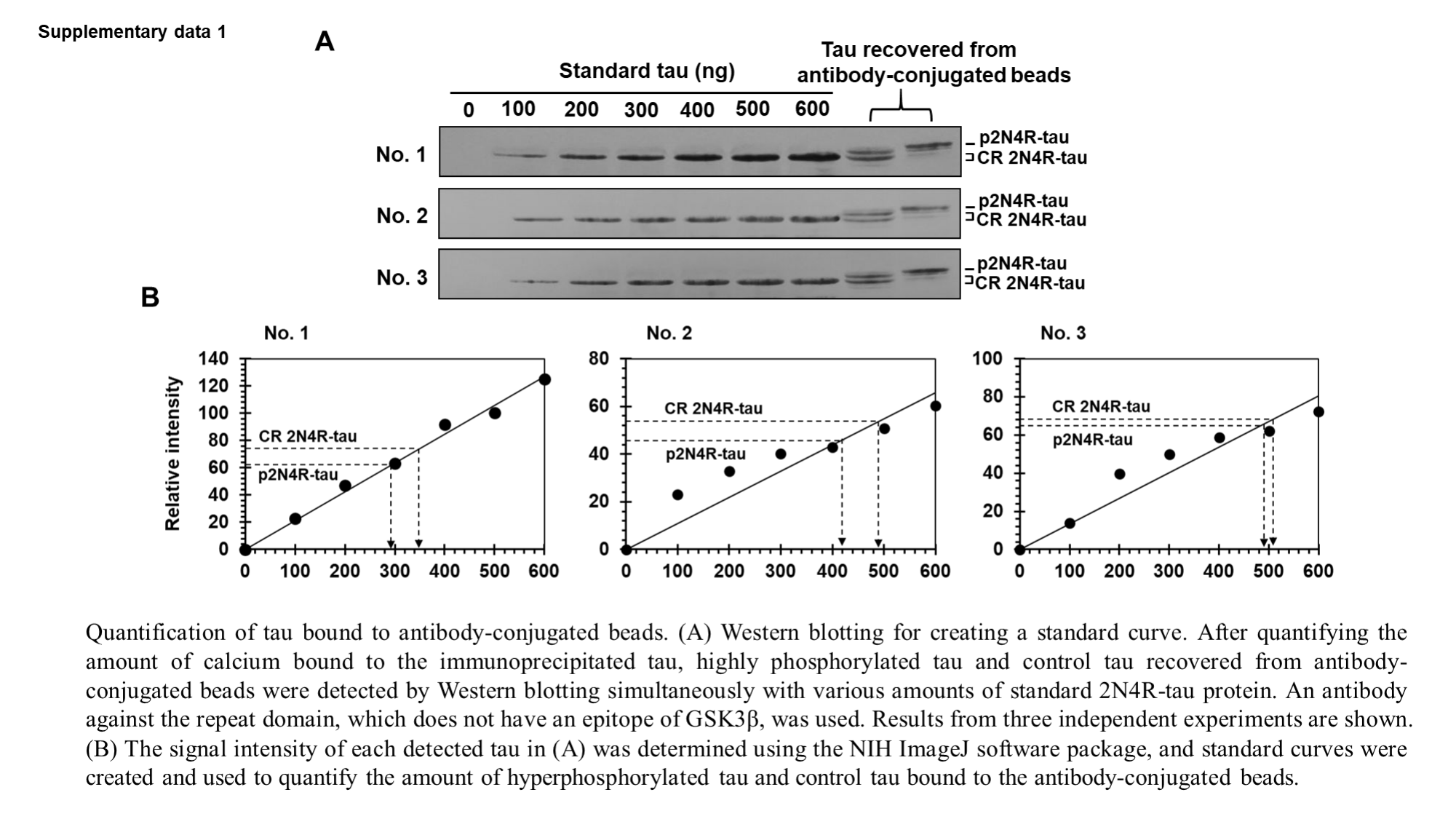

Supplement: Multimedia component 1 [file mmc1.pptx]

## Slide 1
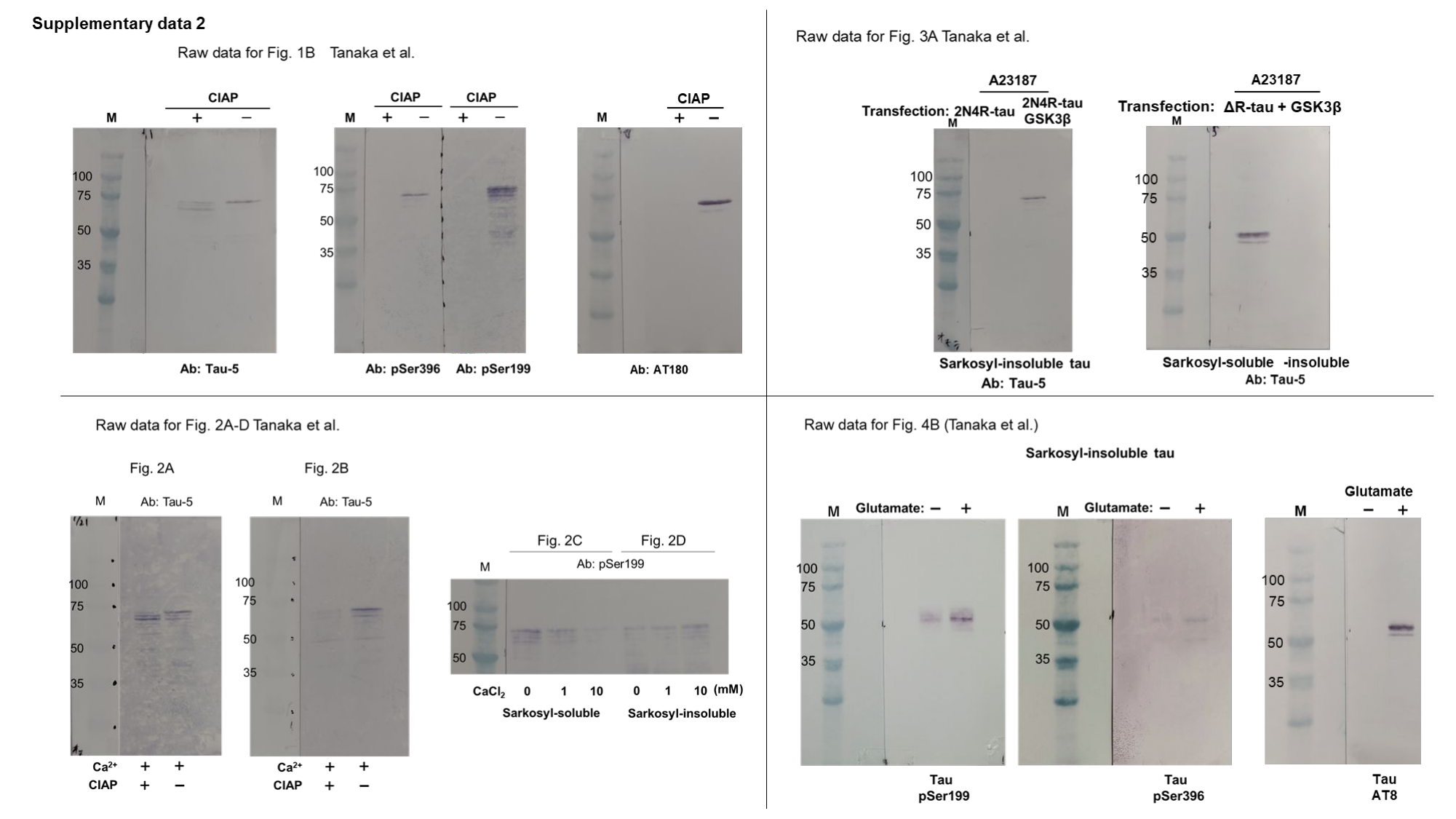

Supplement: Multimedia component 2 [file mmc2.pptx]
